# Reduced bioefficacy of used LLINs against natural populations of *Anopheles gambiae* sensu lato in the centre and east regions of Cameroon

**DOI:** 10.1186/s13071-025-07156-5

**Published:** 2026-01-29

**Authors:** Belinda Claire Kiam, Aline Gaelle Bouopda-Tuedom, Jean Arthur Mbida Mbida, Ibrahima Ibrahima, Charlène Tina Nanssong-Vomo, Luc Abate, Lionel Brice Feufack-Donfack, Brigitte Tumamo Fotso, Sandrine Eveline Nsango

**Affiliations:** 1 Department of Animal Biology, Faculty of Sciences, University of Douala, P.O. Box 24 157, Douala, Cameroon; 2 Malaria Research Unit, Centre Pasteur du Cameroun, P.O. Box 1274, Yaounde, Cameroon; 3 Faculty of Medicine and Pharmaceutical Sciences, University of Douala, P.O. Box 2701, Douala, Cameroon; 4 MIVEGEC, University of Montpellier, IRD, CNRS, Research Institute for Development, 911 Avenue Agropolis, P.O. Box 64501, 34394 Montpellier Cedex, France; 5 Malaria Research Unit, Institut Pasteur du Cambodge, No.5 Boulevard Monivong, P.O. Box. 983, Phnom Penh, Cambodia; 6 Department of Biomedical Sciences, Faculty of Sciences, University of Bertoua, P.O. Box 416, Bertoua, Cameroon

**Keywords:** ITNs, Physical integrity, Bioefficacy, *An. gambiae* (s.l.), Eyang, Bertoua, Cameroon

## Abstract

**Background:**

Long-lasting insecticidal nets (LLINs) are crucial for malaria prevention in Cameroon, yet their operational performance may be compromised because of deterioration of the physical integrity and bioefficacy of nets. This study evaluated LLINs, physical integrity, and bioefficacy following mass distribution campaigns in two regions in Cameroon: East (Bertoua) and Centre (Eyang).

**Methods:**

Household surveys were conducted to assess ITN ownership, usage patterns, and maintenance practices. Net condition was measured using the proportionate hole index (pHI), and bio-efficacy was assessed using WHO cone bioassays against *Anopheles gambiae* (s.s.) (Kisumu strain) and local field mosquitoes.

**Results:**

A total of 55 LLINs from Bertoua and 30 from Eyang were sampled. LLIN ownership was comparable between sites (66.7% in Bertoua vs. 67.9% in Eyang), with a higher usage rate in Bertoua (73.3%) compared to 58.2% in Eyang. In Bertoua, a large percentage of LLINs (59.6%) were too torn, with Olyset Plus being the most common brand. In contrast, Eyang had a lower proportion of torn nets (44%), and households used a combination of different brands, including the Olyset net, Permanet 2.0, and Royal Sentry. Against the susceptible Kisumu strain, Bertoua’s Olyset Plus nets showed optimal efficacy with a 94.6% mortality rate, exceeding the World Health Organization's (WHO) threshold of ≥ 80%. In contrast, nets from Eyang had a 79.3% mortality rate, falling short of the optimal threshold. However, when tested against local field mosquitoes, Olyset Plus (pyrethroid + PBO) showed higher efficacy (mortality rate of 31.8%) than the other brands (7.1% from Olyset net; 18.6% for Permanet 2.0; and 8.6% for Royal Sentry) (*p* > 0.0001).

**Conclusions:**

These findings underscore the crucial importance of proper LLINs maintenance, particularly regarding washing practices. The results also indicate the need to deploy newer generation LLINs to address emerging insecticide resistance and strengthen malaria control efforts.

**Graphical Abstract:**

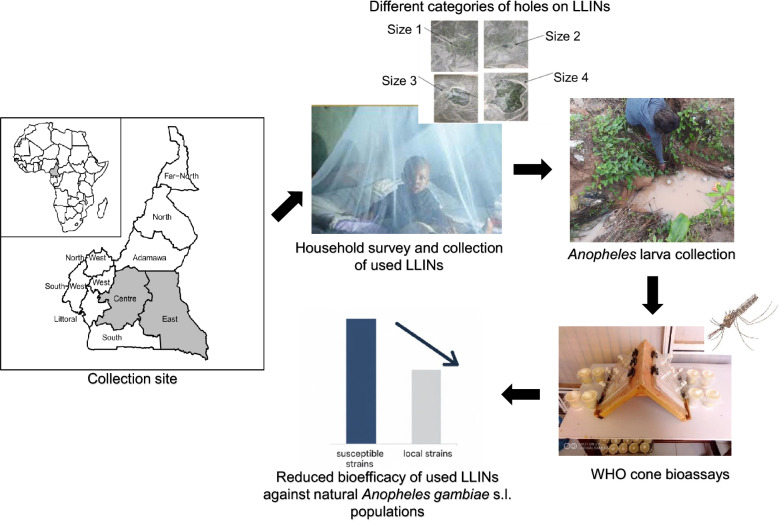

## Background

Insecticide-treated nets are a safe, user-friendly, and effective intervention for protecting against vector-borne diseases [1]. Among these, LLINs are the primary vector control strategy used to combat malaria transmission in endemic areas and have significantly reduced malaria morbidity and mortality, particularly among pregnant women and children under 5 years [2]. LLINs have been a key tool in the fight against malaria, helping to significantly reduce the global burden of the disease. In 2023, there were an estimated 263 million cases and 597,000 deaths from malaria. This represents a substantial decrease in malaria incidence, which fell from 81 per 1000 population in 2000 to 60.4 in 2023. Additionally, numerous global studies have documented a 20–63% reduction in malaria cases and related deaths following LLIN implementation [1, 3].

In Cameroon, LLINs represent the mainstay of malaria vector control. Between 2000 and 2023, substantial efforts have been made to increase LLIN coverage [4], with the proportion of the population sleeping under insecticide-treated nets increasing from 3 to 52%, particularly among young children and pregnant women [5]. Since 2011, Cameroon has undertaken four nationwide mass distribution campaigns, distributing nearly 20 million LLINs free of charge.

Long-lasting insecticidal nets reduce human-vector contact and suppress the reproductive capacity of highly anthropophilic mosquito species, thereby decreasing their lifespan and lowering the sporozoite infection rate [6, 7]. However, despite these achievements, the effectiveness of LLIN has been threatened by several challenges, most notably the emergence and spread of pyrethroid resistance among malaria vectors [8, 9]. Pyrethroids, the major class of insecticides used for LLIN impregnation, have shown declining efficacy due to widespread resistance reported across multiple regions in Cameroon [9, 10].

To address this issue, next-generation “double-action” LLINs are being deployed in malaria-endemic settings [11, 12]. These innovative nets use two strategies: (i) pyrethroid + PBO nets, in which a pyrethroid (e.g., deltamethrin or permethrin) is combined with piperonyl butoxide (PBO), a synergist that counteracts cytochrome P450-mediated metabolic resistance [13–15]; (ii) dual insecticide LLINs, which combine alphacypermethrin with chlorfenapyr, a novel public health insecticide capable of killing up to 75% of mosquitoes and remaining effective for 20 washes [16–18]. Unlike conventional pyrethroid-based nets that target the mosquito’s nervous system, chlorfenapyr-based LLINs disrupt cellular respiration, resulting in immobilization and death [17, 18]. Although the WHO has provisionally recommended these new-generation nets, comprehensive evaluations have been ongoing before full endorsement.

Furthermore, evaluating LLIN durability under operational conditions is essential to improving malaria control strategies. The 2011 WHO guidelines emphasize the need to monitor LLIN longevity, physical condition, and insecticidal effectiveness to guide net replacement timing [19–21]. Field assessments have reported rapid physical degradation of LLINs, with their effective lifespan ranging between 2 and 4 years, or even less in some contexts [9, 22]. This variability is influenced by the nets' fabric quality, housing structure, environmental exposure, and user maintenance practices [23, 24].

The present study evaluated the physical integrity and bioefficacy of nets in Bertoua and Eyang at 1 and 4 years after their distribution.

## Methods

### Description of study areas

**Fig. 1:**
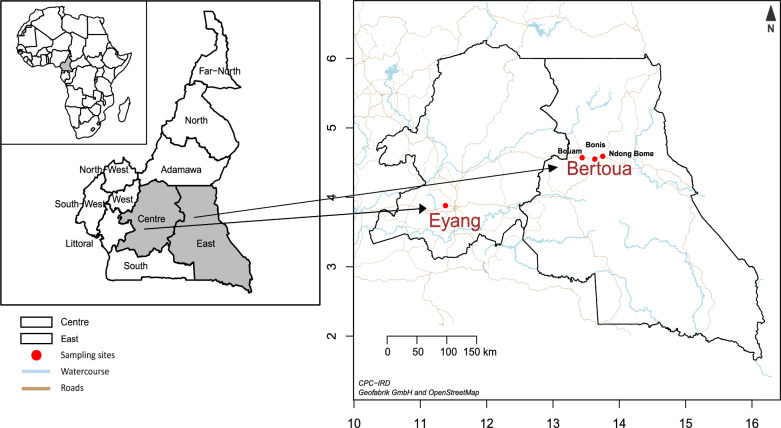
Map showing the different study sites (OpenStreetMap data from Geofabrik GmbH)

The study was conducted in September 2020 in Eyang (3°52′60″N and 11°22′60″E) and in December 2020 in Bertoua (4°34′38″N and 13°41′4″E) (Fig. 1).

Eyang is situated in the commune of Lobo, Centre Region. The commune covers an area of 260 km^2^, with an estimated population of around 700 inhabitants [25]. The area has a four-season equatorial Guinean climate, with an annual rainfall of approximately 133.3 mm/year [25]. Houses are mostly built of permanent materials, and the natural environment is peri-urban. The malaria morbidity in this region is estimated at 30.4 per 1000 inhabitants [26].

Bertoua is located in the East Region, with an estimated area of 100 km^2^ and a population of around 120,000 [27]. The study was conducted in three villages: Bouam, Ndong-Bome, and Bonis I. The climate is equatorial Guinean type with four seasons and annual rainfall of 1500–2000 mm/year. The area is drained by two main rivers: the Lenguengue to the west and the Djadombe to the east, with dwellings built mostly of permanent materials. The malaria morbidity in this region is estimated at 29.7 per 1000 inhabitants [26].

In these localities, LLINs and repellents are frequently used by the populations against *Anopheles* bites [14].

### Bed net collection and household surveys

A systematic randomized household sampling approach was performed in each locality. The process involved selecting a random starting point and then visiting every n-th household to collect and replace nets. Cross-sectional community surveys were also conducted using a structured questionnaire in official languages (English/French) or local dialects. This questionnaire was designed to assess LLIN ownership, usage frequency, maintenance practices (washing and drying methods), age, and type of nets. Household construction materials were also recorded. For this study, net usage was specifically defined as follows: (i) Regular use: if the household used its bed net every night of the week preceding the survey; (ii) Irregular use: if the household only used it for a few nights; (iii) Nothing: if the bed net had not been used for the whole week. The proportion of usage was obtained for all surveyed households where at least one mosquito net was reported to have been used the previous night.

To assess their residual biological efficacy, a total of 85 LLINs were collected from households in both Bertoua and Eyang. From each household, only one net was collected. The sample sizes were unequal, with 55 nets from Bertoua and 30 from Eyang, because of the varying number of eligible LLINs found in the enrolled households. Each collected net was labeled, stored, and then sent for laboratory analysis. To ensure the household remained protected, all the nets collected from participants were immediately replaced with new ones.

Prior to questionnaire administration, participants received an information sheet detailing the study objectives and design. Only individuals who provided written informed consent were enrolled.

### Physical integrity of nets

The physical integrity of the nets was assessed by checking for the presence of holes on each side of the net. The holes were counted and classified into four categories according to WHO [28], size 1 (0.5–2 cm), size 2 (2–10 cm), size 3 (10–25 cm), and size 4 (> 25 cm), and according to the position on the net (roof, top, and bottom). The observations made on each net were used to calculate two indicators:

- The proportion of bed nets with holes, calculated using the formula: 100 × number of bed nets with at least one hole/total number of bed nets inspected [95% CI] [10].
- The proportional hole index (pHI), calculated as the sum of the individual proportional indexes, with each category’s proportional index being the product of the number of holes in that category and the index attributed to its corresponding hole size.

(pHI): (a x number S1) + (b x number S2) + (c x number S3) + (d x number S4).

With a = 1, b = 23, c = 196, and d = 576, a, b, c, and d were estimated from the respective mean areas of the different categories of holes.

### *Anopheles* larva sampling and mosquito identification

*Anopheles gambiae* (s.l.) larvae and pupae were collected from all identified breeding sites within the study areas [29]. Larvae were in tanks and fed with TetraMin Baby^®^ fish food. Emerging pupae were transferred to small dishes and placed in rearing cages until adult emergence. Adult females were maintained on a 10% sugar solution until bioassays were conducted. Morphological identification of adult mosquitoes was performed using standard taxonomic keys [30]. Genomic DNA was extracted from the heat-thorax and legs of adult mosquitoes using a 2% cetylmethylammonium bromide (CTAB) solution [31]. Species identification within the *An. gambiae* complex was performed by multiplex polymerase chain reaction (PCR) [32]. *Anopheles gambiae* (s.l.) mosquitoes aged 2–5 days, female, were used for bioefficacy testing.

### Bio-efficacy of nets

Five pieces, 25 cm × 25 cm from each net side, were cut and wrapped in aluminum foil and stored at 4 °C until the tests were carried out. Bioassays were conducted using the WHO cone test [28].

The residual efficacy of LLINs was evaluated against the laboratory-reared fully susceptible *An. gambiae* (s.s.) Kisumu strain (mortality rate ≥ 98% after 1-h exposure to WHO-impregnated papers) and field-collected *Anopheles coluzzii*, pyrethroid-resistant of the Tibati strain, Adamaoua region of Cameroon [33].

Four cones were connected at their widest opening on each piece of the net. Using a mouth aspirator, five adult non-blood-fed females, *An. gambiae* (s.l.) aged 2–5 days, were introduced into each cone, totaling 20 mosquitoes per piece, and exposed to net samples for 3 min. For each test day, 20 mosquitoes (five per cone) of the same *Anopheles* strain were exposed to an untreated net to serve as a negative control. Similarly, new unused nets of each LLIN type were tested as a positive control. After exposure, mosquitoes were transferred to paper cups covered with a non-insecticidal net, fed with 10% sucrose, and maintained at 27 ± 2 °C and 80 ± 10% relative humidity. Knockdown was recorded 1-h post-exposure, and the mortality was assessed 24 h later. If the mortality in the control was < 10% for a given day, the data were adjusted with Abbott’s formula [34].

### Data analysis

Data collected were entered into an Excel database and transferred to R Studio software version 2023.12.1 and SPSS (Statistical Package for Social Sciences) version 22.0 statistical analysis software for analysis.

The pHI was used to classify the nets into three categories based on WHO recommendations [28]: A net was considered good if pHI was from from 0 to 64, damaged if pHI was from 65 to 642, and torn if pHI was from ≥ 643. The results of efficacy testing of each piece of the net were pooled and used to determine whether the net met the following criteria [35]: optimal efficacy if mortality ≥ 80% or knockdown ≥ 95%; minimal efficacy if mortality ≥ 50% or knockdown ≥ 75%; not effective if mortality < 50% or knockdown < 75%.

The Chi^2^ test was used to compare the proportions of categorical variables, such as net usage and maintenance practices. For normally distributed continuous variables, such as mean mortality and knockdown rates, the Student’s *t*-test was used for comparisons between the two localities. The Kruskal-Wallis H test was used to compare the differences in the hole size of the LLIN collected. Additionally, a post hoc analysis was performed using the Dunnett test to make pairwise comparisons of the mean hole indices between the sites. The significance level was set at *p* < 0.05.

## Results

### LLIN possession and brands

A total of 126 households were enrolled in the study (45 in Eyang and 81 in Bertoua). Of these, 67.5% (*n* = 85/126) households had at least one mosquito net, while 6.4% (*n* = 8/126) reported not sleeping under a net because of either a lack of access or its poor condition. Mosquito net ownership was comparable between the two sites, with a rate of 66.7% (*n* = 30/45) in Eyang and 67.9% (*n* = 55/81) in Bertoua. The nets collected in Eyang had been in use for 4 years, originating from the second mass distribution campaign in 2015. These nets comprised various brands: Olyset net (76.7%, *n* = 23/30), Permanet 2.0 (13.3%, *n* = 4/30), and Royal Sentry (10%, *n* = 3/30). In contrast, all nets collected from Bertoua were Olyset Plus brand and one in use and originated from the third mass distribution in 2019 (Table 1).

**Table 1:** Long-lasting insecticidal net brands in Eyang and Bertoua

Study sites	LLIN brands	Number of nets collected		Insecticide (noncentration)	Source of nets
		N	%		2nd mass campaign
Eyang	PermaNet^®^ 2.0	4	13.3	Deltamethrin (55 mg/m^2^)	
	Olyset^®^ Net	23	76.7	Permethrin (800 ± 200 mg/m^2^)	
	Royal Sentry Net	3	10	Alpha-cypermethrin (261 mg/m^2^)	
Bertoua	Olyset^®^ Plus	55	100	Permethrin (400 ± 100 mg/m^2^ + 2% PBO)	3rd mass campaign

### LLIN use and maintenance

The proportion of net in use was higher in Eyang, with 73.3% (*n* = 22/30) of respondents reporting regular use compared to 58.2% (*n* = 32/55) of respondents in Bertoua. A statistically significant difference was observed between the two sites (*p* = 0.005). Across all net types, 62.3% (*n* = 53/85) of respondents had washed their nets at least once. A statistically significant difference in washing frequency was found between the two sites (*p* = 0.037), with Bertoua reporting a higher rate (65.4%, *n* = 36/55) compared to Eyang (56.7%, *n* = 17/30).

Regarding how the LLINs were washed, most respondents used soap (52.9%), followed by detergent (37.7%). A small number of people (9.4%) used both. For drying, most nets (83.2%) were dried outdoors in direct sunlight, while the remaining 16.9% were dried in the shade (Table 2).

**Table 2:** Long-lasting insecticidal net use and maintenance

Category			Eyang		Bertoua		Total	
			N	%	N	%	N	%
Frequency of bed nets in use	Regular		22	73.3	32	58.2	54	63.5
	Irregular		5	16.7	13	23.6	18	21.2
	None		3	10	10	18.2	13	15.3
	Total		30	100	55	100	85	100
Bed net maintenance	Clean		6	20	8	14.5	14	21.2
	Dirty		24	80	47	85.5	71	83.5
	Total		30	100	55	100	85	100
Total number of washes	Unwashed		13	43.3	19	34.5	32	37.6
	Washed	Washed (1–6)	14	46.7	36	65.5	50	58.8
		Washed (≥ 10)	3	10	–	–	3	3.5
	Total		30	100	55	100	85	100
Type of washing agent	Soap		7	4.1	21	58.4	28	52.9
	Detergents		5	29.4	15	41.2	20	37.7
	Soap and detergent		5	29.4	–	–	5	9.4

% = percentage; regular = if the household used its bed net every night of the week preceding the survey; irregular = if the household only used it for a few nights; nothing = if the bed net has not been used for the whole week

### Physical integrity of LLINs

**Fig. 2:**
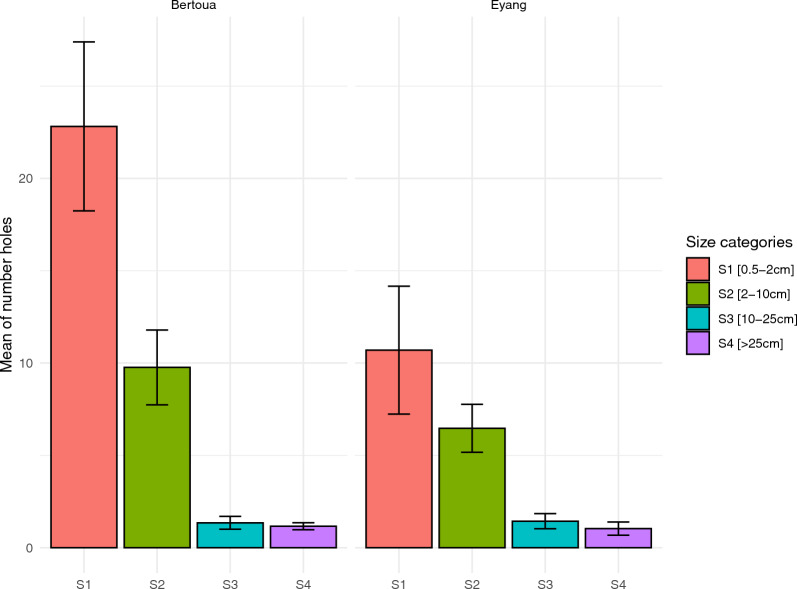
Long-lasting insecticidal net hole proportion by size category in Bertoua and Eyang

A total of 85 LLINs (55 in Bertoua and 30 in Eyang) were assessed for their physical integrity. Most of these nets, 90.6% (*n* = 77/85), had at least one hole. The proportion of holes was higher in Bertoua, 94.5% (*n* = 52/55), compared to Eyang, 83.3% (*n* = 25/30). A total of 2519 holes were found across all nets, including 1930 in Bertoua and 589 in Eyang, with an average of 29.64 holes per net (± 4.5). Bertoua had a significantly higher average of 35 ± 6.5 compared to Eyang nets, which had an average of 20 holes (± 4.1). The holes were categorized into four sizes, with S1 being the most prevalent, 54.5% (*n* = 1373/2519). This was followed by S2 32.9% (*n* = 829/2519), S3 8.8% (*n* = 222/2519), and S4 3.2% (*n* = 95/2519). Most of these holes, 78.3% (*n* = 1973/2519), were located on the lower parts of nets, accounting for 81.8% (*n* = 1578) in Bertoua and 67.1% (*n* = 395) in Eyang (Fig. 2).

The percentage of LLINs in good condition was low across both Bertoua and Eyang (Table 3), 16.4% (*n* = 9/55) and 16.7% (*n* = 5/30), respectively. When examining specific brands, Olyset net and Olyset Plus showed comparable proportions of good-condition nets at 21.8% (*n* = 5/23) and 16.4% (*n* = 9/55), respectively. Notably, no Permanet 2.0 or Royal Sentry net was found in good condition (Table 3).

**Table 3:** Long-lasting insecticidal net status in Bertoua and Eyang

Category	Bertoua		Eyang						Total	
	Olyset Plus		Olyset Net		PermaNet 2.0		Royal Sentry			
	N	%	N	%	N	%	N	%	N	%
LLINs collected	55	64.7	23	27.1	4	4.7	3	3.5	85	100
Number of nets with one hole	52	94.5	18	78.3	4	100	3	100	77	90.6
Good (0 ≤ pHI ≤ 64) (%)	9	16.4	5	21.8	–	–	–	–	14	16.5
Damaged (64 < pHI ≤ 642) (%)	15	27.3	3	13.1	3	75	3	100	29	34.1
Torn pHI > 642 (%)	31	56.4	10	43.5	1	25	0	0	42	49.4
Average proportional hole index (pHI)	1264.23077		1037.633		937.217		371.32		–	

### Bio-efficacy of LLINs

A total of 85 LLINs were tested for bio-efficacy using susceptible Kisumu strain mosquitoes. For each side of every net, 20 mosquitoes were used. Mortality rates ranged from 0 to 100%. The median (interquartile range, IQR) for KD60 in Bertoua was 96.5% (92–100%), and for overall mortality, it was 100% (98–100%). In Eyang, the median (IQR) for KD60 was 78.4% (56–90%), and for overall mortality, it was 85.2% (69–100%). The differences between sites for KD60 and overall mortality were statistically significant (*p* = 0.047 and *p* < 0.001, respectively). Overall, nets from Bertoua showed optimal efficacy against the susceptible Kisumu strain, while those from Eyang presented minimal efficacy: Olyset net had an efficacy of 74.9% [95% CI 67.8–80.1%], Permanet 2.0 88.4% [95% CI 79.5–93.4%], Royal Sentry 99.3% [95% CI 95.8–99.5%], and Olyset Plus 94.6% [95% CI 91.1–96.6%] (Table 4). However, a significant difference in efficacy was observed between LLINs from Bertoua (94.6%, [95% CI 91.1–96.6%]) and Eyang (79.3%, [95% CI 74.4–83.3%]) (*p* < 0.0001).

**Table 4:** Mortality and knockdown rates of susceptible strain against LLINs collected

Mosquito species	LLIN brands	Number of tested mosquitoes	Number of dead mosquitoes	Kd_60_ (%) 95% IC	Mortality rate (%) [95% CI]	Net status
Susceptible strain mosquito (*Anopheles gambiae* s.s.)	Olyset net	2289	1715	81.7 [74.2–89.2]	74.9 [67.8–80.1]	Minimal efficacy
	Permanet 2.0	398	352	94.2 [91.8–96.6]	88.4 [79.5–93.4]	Minimal efficacy
	Royal Sentry	299	297	98.9 [96.4–101.4]	99.3 [95.8–99.5]	Optimal efficacy
	Olyset Plus	5489	5191	92.2 [88.6–95.8]	94.6 [91.1–96.6]	Optimal efficacy

**Fig. 3:**
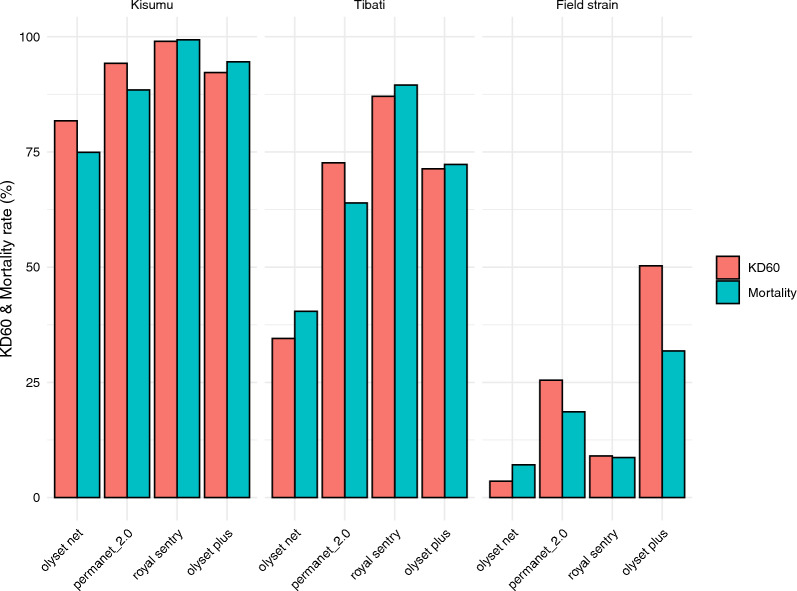
Knockdown (KD60) and mortality rates of susceptible and pyrethroid-resistant *Anopheles gambiae* (s.l.) mosquito populations

When tested with field-collected *Anopheles* strains, LLINs from both localities demonstrated a marked decline in efficacy. Furthermore, LLINs from Eyang consistently showed lower efficacy against field strains compared to those from Bertoua, as evidenced by the significant difference in mortality rates (*p* < 0.0001). Notably, the nets from both sites failed to reach the 50% mortality threshold required for minimal effectiveness (Fig. 3).

### Effect of washing and use of soap on LLIN

**Fig. 4:**
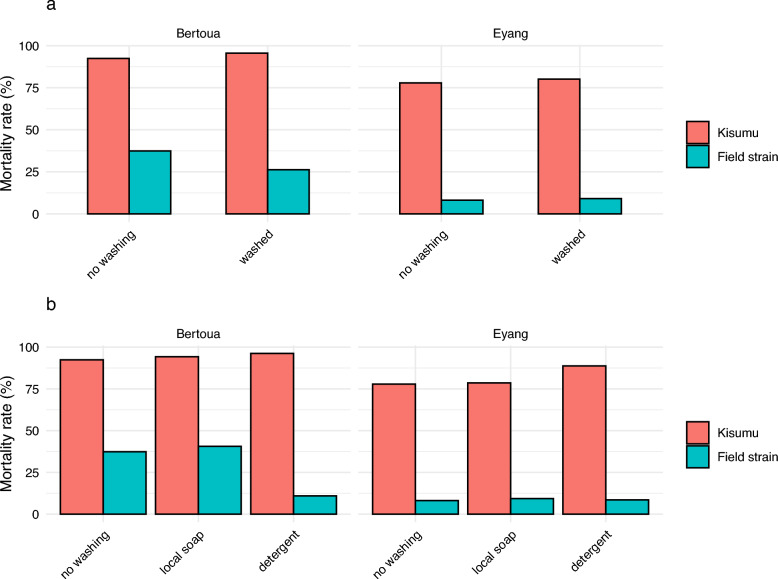
Effects of washing and use of soap on LLIN effectiveness. **a** Bioefficacy of washed net compared to unwashed nets. **b** Bioefficacy of washed nets with soap types

Against the susceptible Kisumu strain, washing nets showed slightly higher efficacy than unwashed nets in Bertoua (95.5% vs. 92.4%, [95% CI 80.7–95.1%], *p* = 0.2) and in Eyang (80.2% vs. 77.9%, [95% CI 72.7–85.4], *p* = 0.9), although no significant difference was observed (Fig. 4a).

Against the susceptible Kisumu strain, LLINs washed with detergent demonstrated similar efficacy to those washed with local soap in both locations (96.2% vs. 94.23% in Bertoua and 88.7% vs. 78.6% in Eyang). However, against field strains, LLINs washed with local soap in Bertoua showed significantly higher mortality rates than those washed with detergent (40.6% vs. 10.9%, *p* = 0.007). No significant difference in mortality rates was observed between washing methods in Eyang (9.35% vs. 8.58%, *p* = 0.7) (Fig. 4b). These results indicate that although used LLINs may retain some efficacy against susceptible strains regardless of washing method, their effectiveness against field strains is highly sensitive to the type of washing agent used. The significant decline in efficacy with detergent washing suggests that the harsh surfactant properties of detergents may be more effective at stripping the insecticide from net fibers.

### Species composition of field-collected *Anopheles* mosquitoes

A molecular analysis was conducted on 238 *An. gambiae* (s.l.) (112 from Bertoua and 126 from Eyang). The results showed that in Beroua, the population consisted of 70.5% *An. gambiae* s.s. and 29.5% *An. coluzzii*. In Eyang, the population was made up of 72.2% *An. gambiae* s.s. and 21.4% *An. coluzzii*, with a small proportion of 6.3% hybrids also present. No hybrids were detected in Bertoua.

## Discussion

This study aimed to assess the physical integrity and bioefficacy of LLINs distributed during mass campaigns in two Cameroonian localities (Bertoua and Eyang). LLIN ownership was suboptimal in both sites (67.5%), falling below the 80% threshold recommended by the WHO for achieving universal coverage [36–39]. This could be attributed to households reporting that they did not receive LLINs during the campaign or had abandoned them because of excessive wear or ageing. Similar findings were reported by Nopowo et al. in Ayos, a locality in southern Cameroon [10].

In Eyang, LLINs of various brands were found, including Olyset net, Permanet 2.0, and Royal Sentry, all treated with pyrethroids. In contrast, only Olyset Plus nets (pyrethroid + PBO) were found in Bertoua. This distribution pattern reflects the differentiated strategy adopted by the National Malaria Control Programme (NMCP) during the third nationwide campaign [40]. In response to widespread pyrethroid resistance, particularly in high-transmission zones like Bertoua, the NMCP prioritized the distribution of PBO-based LLINs to enhance vector control efficacy and delay further resistance development, especially among vulnerable populations [14, 22].

Despite distribution efforts, LLIN usage remained below the recommended threshold, with 73.3% in Eyang and 58.2% in Bertoua, both below the 80% WHO recommendation for community protection [41]. This reduced usage may result from discomfort associated with net use, including heat, feelings of suffocation, or alternative uses such as fishing [42, 43]. The variation between sites may also be explained by seasonal differences during sampling: the end of the rainy season in Eyang and the beginning of the dry season in Bertoua. Studies in Ghana and Burkina Faso have shown lower net usage during dry seasons due to reduced mosquito nuisance and higher indoor temperatures [44, 45]. Moreover, the texture of the Olyset Plus nets, with a less rigid mesh, may contribute to discomfort, potentially discouraging use. These findings underscore the importance of continuous community sensitization campaigns to encourage consistent LLIN use and address behavioural and contextual barriers [1].

LLIN physical condition plays a critical role in protection. Nearly half of the nets examined had a proportional hole index (pHI) ≥ 643, classifying them as torn and thus likely ineffective in preventing mosquito bites. This is consistent with findings from Kenya and Senegal, where significant LLIN deterioration was reported within months of use [46, 47]. Conversely, 12.9% of nets were in good condition, suggesting recent acquisition or proper handling. Most damage was concentrated in the lower parts of the nets, frequently handled areas during entry and exit, consistent with findings from Ghana [19, 21]. The higher proportion of damaged nets in Bertoua may be linked to housing conditions (e.g., mud walls, wooden frames, bamboo beds) and maintenance behaviours (frequent washing, drying practices, and handling). Additionally, net brand composition and fiber type appear to influence durability: the finer fibers of Olyset Plus nets in Bertoua may contribute to their higher vulnerability to damage compared to the more robust Olyset net in Eyang [48–50].

This physical damage is particularly concerning in a high-resistance area like Bertoua, where a hole provides an unimpeded pathway for mosquitoes that are no longer susceptible to the remaining insecticide [9, 51]. This underscores the importance of a targeted distribution strategy using new-generation nets (e.g., PBO) in such areas. While PBO nets are highly effective, their high cost must be considered against their superior efficacy. It may be more cost-effective to invest in a continuous, targeted net replacement supply chain rather than to rely solely on mass campaigns. The impact of washing on net efficacy also highlights the need for improved community education on proper net maintenance to prolong the useful life of these tools.

Bioefficacy evaluation of nets revealed low mosquito mortality rates of 50% for LLINs collected in both sites when tested against field *An. gambiae* (s.s) *and An. coluzzii* mosquito populations. This reduced efficacy is related to factors such as the frequency of washing, inappropriate soap use, and poor environmental hygiene. Many nets were visibly soiled with dust or urine, giving them a more or less black color, forming residues that can hinder insecticide diffusion across net fibers, a phenomenon known as fouling. Previous studies have demonstrated that smoke, dust, and organic matter may bind or coat insecticidal molecules, thereby compromising their net efficacy [52–54]. Notably, unwashed nets showed lower mortality against the susceptible Kisumu strain than nets that had been washed at least once, possibly due to better release of the insecticide after initial washing. However, repeated washing with soap or detergent, as well as drying nets under sunlight, has been shown to degrade the insecticide, further reducing LLIN bioefficacy [46, 53].

Nets collected during this study demonstrated optimal efficacy (mortality ≥ 95%, as per WHO guidelines) against the susceptible laboratory strain *An. gambiae* “Kisumu” but significantly reduced performance against the resistant *An. coluzzii* "Tibati" strain and field populations. Mortality rates differ significantly between LLIN types: nets from Bertoua (Olyset Plus) induced higher mortality than those from Eyang (Olyset net, Permanet 2.0, Royal Sentry). The enhanced efficacy of Olyset Plus is attributed to the presence of PBO, which inhibits detoxification enzymes in resistant mosquitoes, thereby restoring the potency of pyrethroid insecticide. These findings support previous studies by Nkahe et al. [14] and are consistent with research by Pennetier et al. [55–57], demonstrating improved performance of Olyset Plus against multi-resistant *An. gambiae* (s.l.) populations. While PBO nets offer a significant advantage, it is crucial to recognize their limitations. The efficacy of PBO is not permanent, as the synergist can degrade over time and with repeated washing, potentially leading to a resurgence of pyrethroid resistance. Moreover, the long-term effectiveness of these nets may be threatened by the emergence of new resistance mechanisms or behavioural changes in mosquito populations [15].

## Conclusions

While the distribution of LLINs is effective for malaria vector control, this study found a significant reduction in their physical integrity and bioefficacy due to inappropriate washing and maintenance practices, highlighting the need for more frequent net replacement, especially in high-usage areas. Furthermore, the differentiated performance of various net brands and types (PBO vs. pyrethroid-only) underscores the importance of a targeted distribution strategy for new-generation nets, such as PBO LLINs, in areas with confirmed pyrethroid resistance. To ensure long-term effectiveness and prevent future challenges, robust community education is needed to promote proper net usage and maintenance. It is also advisable to conduct long-term durability trials of new-generation nets under operational conditions and investigate the cost-effectiveness of different net replacement and maintenance strategies.

## Data Availability

All data generated or analyzed during the current study are included in this published article.

## Abbreviations

IRS: Indoor Residual Spraying
LLINs: Long Lasting Insecticide Net
OCEAC: Organisation pour la Coordination de la lutte contre les Endémies en Afrique Centrale
PBO: Piperonyl Butoxide
pHI: Proportionate Hole Index
NMCP: National Malaria Control Program
WHO: World Health Organization
